# USAEME-GC/MS Method for Easy and Sensitive Determination of Nine Bisphenol Analogues in Water and Wastewater

**DOI:** 10.3390/molecules27154977

**Published:** 2022-08-05

**Authors:** Dariusz Kiejza, Urszula Kotowska, Weronika Polińska, Joanna Karpińska

**Affiliations:** 1 Doctoral School of Exact and Natural Sciences, University of Bialystok, Ciołkowskiego 1K St., 15-245 Białystok, Poland; 2 Department of Analytical and Inorganic Chemistry, Faculty of Chemistry, University of Bialystok, Ciołkowskiego 1K St., 15-245 Białystok, Poland

**Keywords:** bisphenol analogue, liquid–liquid microextraction, acylation, gas chromatography-mass spectrometry, municipal wastewater

## Abstract

A new, simple and sensitive method for isolating nine compounds from the bisphenol group (analogues: A, B, C, E, F, G, Cl_2_, Z, AP) based on one-step liquid–liquid microextraction with in situ acylation followed by gas chromatography-mass spectrometry was developed and validated using influent and effluent wastewaters. The chemometric approach based on the Taguchi method was used to optimize the main conditions of simultaneous extraction and derivatization. The recoveries of the proposed procedure ranged from 85 to 122%, and the repeatability expressed by the coefficient of variation did not exceed 8%. The method’s limits of detection were in the range of 0.4–64 ng/L, and the method’s limits of quantification ranged from 1.3 to 194 ng/L. The developed method was used to determine the presence of the tested compounds in wastewater from a municipal wastewater treatment plant located in northeastern Poland. From this sample, eight analytes were detected. Concentrations of bisphenol A of 400 ng/L in influent and 100 ng/L in effluent were recorded, whereas other bisphenols reached 67 and 50 ng/L for influent and effluent, respectively. The removal efficiency of bisphenol analogues in the tested wastewater treatment plant ranged from 7 to approximately 88%.

## 1. Introduction

Bisphenols (BPs) are chemical compounds that contain two phenolic groups in their structure. They are linked to either aliphatic or aromatic groups and, occasionally, to sulfur or fluorine heteroatoms. The best known and most widely used compound in this group is bisphenol A (BPA), a substance first obtained at the end of the 19th century by Alexander Dianin [1]. BPA is easily and inexpensively synthesized from phenol and acetone, which contribute to its wide industrial use. BPA is the main component in the production of polymers, such as polycarbonate plastics and epoxy resins [2]. It has been widely used in many products, including water pipes, food containers, can linings, kitchenware, medical equipment, toys, sealants, thermal paper, electronics and even dental fillings [3,4]. It is estimated that the world’s annual production of BPA is at least five million tons [5]. BPA can migrate from packaging to food and water, making diet the main route of human exposure to this compound. The production, use and recall of materials containing BPA cause the release of this compound into the environment. The release of BPA into the environment is concerning, as its estrogenic activity may cause adverse effects on the intact physiology of organisms. This is an undesirable outcome due to the harmful effect of this compound on organisms, especially through endocrine disturbance of the hormone balance. Interestingly, even in the 1930s, before the rapid development of the plastics industry, the estrogenic properties of this compound were known [6]. Detailed studies have shown that BPA might be related to a wide range of adverse health effects, including diabetes, obesity, reproductive disorders, cardiovascular diseases, breast cancer and birth defects [7].

BPA is detected in municipal and industrial wastewater in concentrations up to mg/L, as well as in leachate from landfills, whereas concentrations of tens of µg/g are found in sewage sludge. Incomplete sewage treatment, sewage sludge management and inadequate protection of municipal landfills are the main sources from which BPA penetrates into surface and ground waters, where BPA concentrations can be as high as several dozen µg/L [8,9,10,11,12]. This compound is also common in soil and air samples, as well as in the tissues of living organisms [13,14]. Biomonitoring studies show that human exposure to BPA is continuous and rapidly growing, resulting in its presence in the body of every modern human being [15]. Due to its proven toxic effect on organisms, restrictions on its application have been set by the European Commission, the U.S. Environmental Protection Agency and Health Canada [16]. New research on BPA’s impact on organisms both confirms and broadens the knowledge about the harmfulness of this compound, which means that the existing restrictions are constantly being expanded [17]. Legal restrictions and increased public concerns have led to the replacement of BPA in industrial production with other compounds of a similar structure, especially bisphenol analogues, which exhibit the same or improved plasticizer properties. Although alternative BPs were originally thought to be less toxic than BPA, recent reports have shown that they have similar or even greater adverse effects than BPA [4,17]. BPs show comparable biological activities to BPA, including the potential for hormonal disruption, toxicity and genotoxicity. Some compounds from this group may have even higher estrogenic activity than BPA [18,19]. Due to their increasing use, these analogues have been detected in different environmental and biological samples [20,21,22,23]. However, knowledge about their spread and how they enter the environment is still insufficient. Research in this area is very complicated due to extremely low BP concentrations at the ng/L–µg/L levels, complicated matrix composition and the lack of standard analytical methods. The most common method for isolating BPs from various types of matrices is solid-phase extraction (SPE) [22,24], whereby the determination of these compounds is mainly carried out by liquid chromatography with tandem mass spectrometry (LC-MS/MS) [22,24,25,26]. A wide selection of SPE sorbents and LC stationary phases allow for the development of both sensitive and selective methods of determination. However, the use of SPE-LC-MS/MS procedures is associated with many difficulties. First, SPE is a time-consuming, multistep procedure that usually requires large volumes of samples and organic solvents (high costs, environment pollution). Secondly, SPE columns and sorption beds are normally made of polymers, which carry the risk of contamination of samples with BPs derived from them. Another problem is the low availability of LC-MS/MS devices in standard environmental laboratories and the high cost of such determinations, which is of considerable importance, especially for countries with lower levels of economic development. Therefore, the search for new solutions in the field of BP determination remains an analytical problem. Gas chromatograph with a single mass spectrometer (GC-MS) is an apparatus commonly found in various types of laboratories. GC-MS determinations are simple, more cost-efficient and environmentally friendly because they do not require the use of expensive and toxic solvents. The main issue with this technique is the need to include a derivatization step in the analytical procedure. The main derivatization method used to adapt analytes that are excessively polar and have low volatility to GC determinations is silylation with trimethylsilyl reagent (TMS), *N*,*O*-bis(trimethylsilyl)trifluoroacetamide (BSTFA) or other reagents. This type of reaction requires anhydrous conditions and a multistage procedure [27]. This method of derivatization is not recommended for compounds from the BP group due to the risk of overestimating the obtained results [28].

The aim of the presented work was to develop a new, simple methodology based on ultrasound-assisted emulsification microextraction (USAEME) with in situ derivatization by acetic anhydride and GC–MS for simultaneous determination of nine BPs in water and municipal wastewater: bisphenol F (BPF), bisphenol E (BPE), bisphenol A (BPA), bisphenol C (BPC), bisphenol B (BPB), bisphenol G (BPG), bisphenol Cl_2_ (BPCl_2_), bisphenol Z (BPZ) and bisphenol AP (BPAP). To the best of our knowledge, several BPs (except BPA, BPF and BPS) have, to date, never been simultaneous isolated from environmental samples using the USAEME technique, and derivatization with acetic anhydride has not been used for their determination. The USAEME-GC/MS method has been used for the determination of bisphenols in other types of samples, such as thermal paper, toys, baby utensils and beverages [29,30]. Considering the multitude of bisphenol analogues, it was advisable to develop a method for the simultaneous determination of the largest possible number of the most common analogues in raw and treated sewage, which may be one of the main sources of environmental exposure to these compounds.

## 2. Methods

### 2.1. Chemicals and Solutions

Analytes: BPF, BPE, BPA, BPC, BPB, BPG, BPCl2, BPZ and BPAP with nominal purity of analytical standards and chromatography-grade chlorobenzene, which was used as extraction solvent, were obtained from Sigma Aldrich (Steinheim am Albuch, Germany). Table 1 lists the systematic names, structures and properties of the tested compounds, including the negative logarithm of the acid dissociation constant (pKa) and the logarithm of the *n*-octanol/water partition coefficient (log Kow). Disodium hydrogen phosphate (buffering salt) and acetic anhydride (derivatizing agent) of 99.5% purity were purchased from the same company. Methanol of LC-MS purity was obtained from Merck (Darmstadt, Germany).

Standard solutions of bisphenols with a concentration of 1 mg/mL were prepared by dissolving the appropriate amount of the substance in methanol. Prepared stock solutions were stored at −20 °C for a maximum of 2 weeks. Working solutions were prepared by diluting the standard solutions and were then stored in a freezer for no longer than 1 week. Milli-Q water (Millipore, Burlington, MA, USA) was used both to optimize the process and for the calibration curve. BPs solutions for extraction optimization and for standard curves were prepared immediately before the examination by dissolving the appropriate volume of the standard solution in either Milli-Q water or in wastewater.

### 2.2. Wastewater Samples

Influent and effluent wastewater samples were collected in a municipal wastewater treatment plant (WWTP) in northeast Poland. Processes conducted in the WWTP include mechanical and biological purification without tertiary treatment. In the mechanical stage, grates, sieves, sand traps, settling tanks and grease separators are used. Biological purification is based on the use of conventional activated sludge (CAS) in the flow system. Effluents are discharged into a local river that is part of the Vistula River catchment system within the Baltic Sea drainage basin. The WWTP treats wastewater for a population of 300,000, with an average daily flow of 70,000 m^3^. This treatment efficiency meets the effluent standards required by Polish legislation for a plant of this size. Average daily wastewater samples were collected using glass samplers and placed into glass bottles that had been previously rinsed with the sample (in triplicate). To determine BPs, portions of the samples were filtered through a 0.45 um pore membrane and then acidified with HCl to a pH = 2 to stop the action of the microorganisms. To determine general pollution parameters, the following were determined in accordance with American Public Health Association (APHA) guidelines: chemical oxygen demand (COD), biological oxygen demand (BOD), total nitrogen (TN) and total phosphorus (TP) [31]. The pH and electrolytic conductivity (EC) were measured using a pH/conductivity meter (CPC-505 Elmetron, Zabrze, Poland).

### 2.3. Extraction Procedure

Isolation of the analytes was performed using the USAEME technique, for which the appropriate amount of buffering salt (disodium hydrogen phosphate) and water solution of BPs were introduced to a 25 mL volumetric flask and shaken to dissolve the salt. Then, 5 mL of the solution was taken from the flask and transferred to a conical tube, to which 60 µL of chlorobenzene and 225 µL of acetic anhydride were added. The mixture was then shaken by hand for 10 s and sonicated for 5 min in a Sonorex Digitec 102H ultrasonic water bath (Bandelin, Berlin, Germany). The resulting emulsion was then centrifuged at 4000 rpm in a MPW-250 Med. Instruments (Warszawa, Poland) centrifuge. The collected extract was transferred by a 50 µL Hamilton syringe (Reno, NV, USA) to a glass insert, placed in chromatographic vials and subjected to GC-MS analysis.

### 2.4. GC-MS Analysis

Determination of BPs was performed by gas chromatography with mass spectrometry (GC-MS). The chromatographic analysis was carried out using a 7890B gas chromatograph with an electronic pressure control coupled with a mass-selective detector 5977A (electron impact source and quadrupole analyzer) from Agilent Technologies, Santa Clara, CA, USA. This device was equipped with an HP-5MS column (5% phenylmethylsiloxane) with dimensions of 30 m × 0.25 mm × 0.25 µm film thickness. Helium (99.999%) at a constant flow rate of 1.0 mL/min was used as a carrier gas. An injector worked in splitless mode at a temperature of 250 °C. The oven operated according to the following temperature program: starting temperature, 130 °C for 3 min; then, the temperature was raised by increments of 30 °C/min until reaching 250 °C after 4 min; then, the temperature was raised at a rate of 20 °C/min until reaching the final temperature of 310 °C. The system operated at this final temperature for 5 min, making the total analysis time 19 min. The electron impact source temperature was 230 °C, with an electron energy of 70 eV. The quadrupole temperature was 150 °C, and the GC interface temperature was 280 °C. The MS detector was set to work in selected ion monitoring (SIM) mode. To select the ions for monitoring, the spectra of BP acyl derivatives were first recorded in scan mode. The chromatogram registered the BP mixture in scan mode; the obtained mass spectra of acyl derivatives are shown in Figures S1 and S2 (Supplementary Materials). The *m*/*z* measured in the BP mass spectra signals was 84 units higher the molecular weight of the tested compounds, which confirms the acylation reaction. This is due to the substitution of two hydrogens in the phenolic OH groups with acyl groups of 42 mass units each. Table 1 includes the retention times of BPs, as well as the *m*/*z* used for analyte derivative monitoring (quantification ions are printed in bold font). 

## 3. Results and Discussion

### 3.1. Extraction Solvent Selection

**Figure 1:**
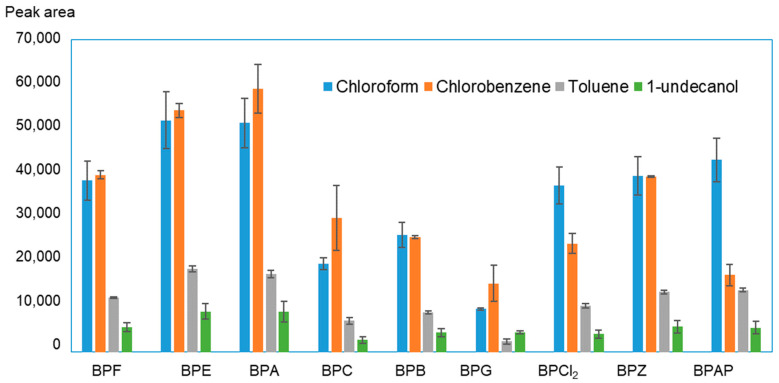
Influence of type of solvent on BP extraction efficiency.

To optimize the extraction process, a 100 µg/L concentration of bisphenols was used in the water solution. The choice of the extractant was preceded by examining the influence of four solvents—chloroform, chlorobenzene, toluene and 1-undecanol—on the extraction efficiency. The results of the examination are shown in Figure 1. Based on the obtained data, it can be concluded that 1-undecanol and toluene provide the smallest analyte surface areas, i.e., the lowest extraction efficiency. The best results were recorded when chloroform and chlorobenzene were used for analyte isolation. Both chloroform and chlorobenzene were previously used as extractants in BPA isolation by liquid–liquid microextraction [11,32,33,34]. Chlorobenzene has, so far, only been used a few times in USAEME for the determination of benzotriazoles, triazine herbicides in environmental water [35,36,37]. For BPs, the effect obtained for these two solvents is very similar. For BPCl_2_, BPZ and BPAP, chloroform achieves better results, whereas for BPF and BPE, chlorobenzene is the better extractant. Chlorobenzene obtained more precise results and was therefore chosen as the solvent to be used in further research.

Chlorobenzene is an organic solvent with a density greater than that of water. Due to its low solubility in water (0.4 g/L at 20 °C), chlorobenzene creates a stable emulsion that not only increases the contact surface of the analytes with the extractant but also enables easier transfer of the analyte to the organic phase. It is easy to use because after centrifugation, solvents with a density greater than that of water accumulate at the bottom of the vessel, making it easier to collect the extract.

### 3.2. Design of Experiments: Chemometric Optimization of the Volumes of Extractant, Derivatizing Reagent and Salt Concentration

The design of experiments (DoE) approach based on the Taguchi method was used to simultaneously investigate the influence of the extractant, derivatizing reagent volume and salt concentration on BP extraction efficiency. This simple method proposed by Genichi Taguchi in the 1980s allows for fast and simple selection of the appropriate process or experimental conditions with the minimum number of experiments performed [29,38,39,40,41]. The main advantage of this method is the use of orthogonal arrays, which balances the influence of individual factors on the response. An L9 orthogonal array with three factors and three levels was selected. The factors that were selected for optimization were as follows: volume of extractant (60, 80, 100 µL), volume of derivatization reagent (125, 175, 225 µL) and salt concentration (0, 2, 4%). The total number of experimental runs was nine. The following lines of the plan represent experiments that were performed in random sequence. Statistical analysis and evaluation of the influence of input factors on extraction efficiency was performed using Minitab^®^ 19 statistical software (Minitab, LLC, State College, PA, USA). 

According to the Taguchi method, the measure of the influence of uncontrollable factors on the response (in this case, the peak area) is the signal-to-noise ratio (SNR). SNR maximization was adopted as the criterion for determining the extraction efficiency, with a higher measure indicating better efficiency [40]:

(1) $$\frac{S}{N}=-10\;\log\left(\frac{1}{n}\overset{n}{\underset{i=1}{\sum }}\frac{1}{y_{i}^{2}}\right)$$

where *n* is the number of experiment runs, and *y_i_* is the output value (peak area) for each *n*th experiment. The higher the signal-to-noise ratio, the more negligible the noise power. Table 2 presents the calculated SNR for each row corresponding to the subsequent experiments.

**Figure 2:**
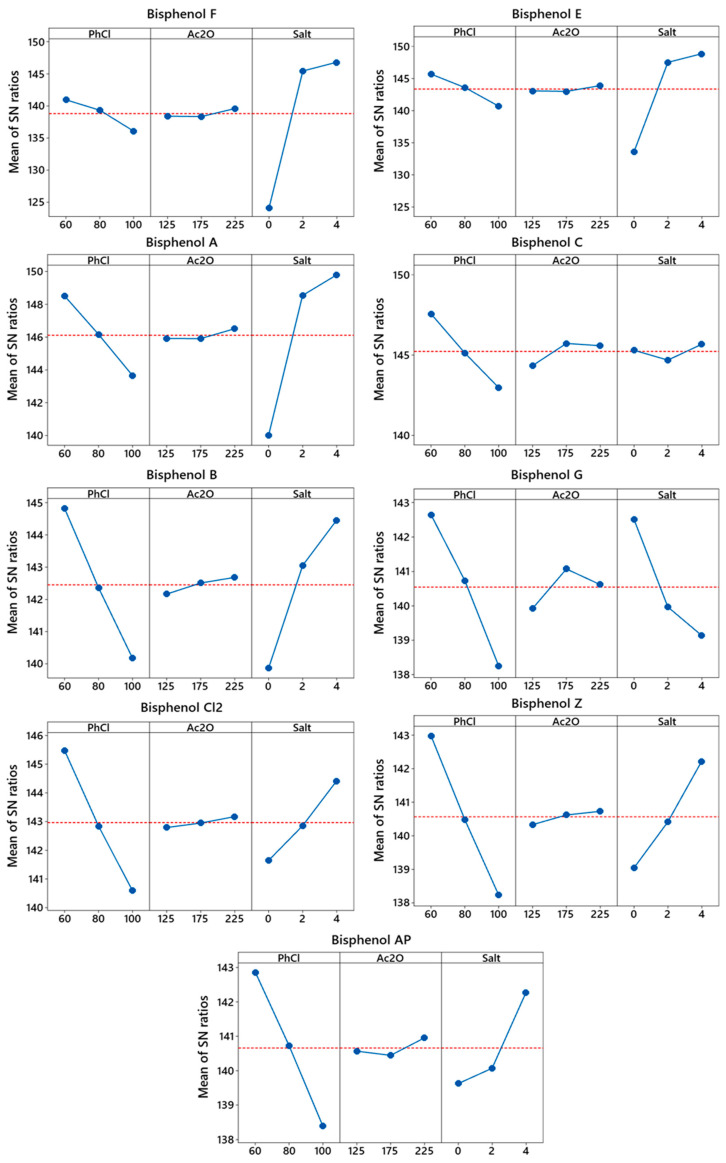
Effect of considered factors on mean SN ratio. Signal-to-noise criterion: larger is better.

The calculated SNR for each factor helps determine how a given input contributes to maximization of the response (Tables S1–S9, Figure 2). For most BPs, the volume of chlorobenzene has the greatest impact on extraction efficiency. This is due to the concentration or dilution effect of the sample and, in turn, a change in the enrichment factors. 

The factors that had the greatest impact on the BP extraction process were identified by analyzing the variance of signal-to-noise ratios. Results of ANOVA for BPA are presented in Table 3. There are eight degrees of freedom in total, with each factor possessing two degrees of freedom. The values of the F test at significance levels of α = 0.1, 0.05 and 0.01 were 3.11, 4.46 and 8.65, respectively. The calculated F parameters are greater for the variables of chlorobenzene and salt, whereas for acetic anhydride, the value of F is lower. It therefore follows that the volume of the derivatizing reagent has a statistically insignificant effect on the extraction yield. Only in the case of BPC (Table S11) does acetic anhydride statistically contribute to the separation efficiency. In the case of BPA, salt has the largest percentage contribution (81.75) in the model. Analysis of the obtained results leads to the conclusion that the obtained model explains 96% of response variability, whereas only 4% remains unexplained.

The applied criterion of the signal-to-noise ratio proved that the efficiency of extraction allowed for the quick selection of the optimal conditions for the simultaneous isolation of the tested analytes. In this study, extraction of bisphenols using 60 µL of chlorobenzene, 225 µL of acetic anhydride and 4% salt with an emulsification time of 5 min allowed for the highest separation efficiency of the tested bisphenols. The predicted SNR for each BP is close to the experimental SNR (Table 4). The consistency of these results shows that the model largely explains the influence of the input values on the response.

#### 3.2.1. Effect of Extractant Volume

In the present study, we examined the effect of chlorobenzene volume on the efficiency of BPS extraction. We found that the SN ratio strongly depended on the volume of the extractant used and that increasing the volume of chlorobenzene reduces the enrichment factor. For all BPs, the optimal volume of chlorobenzene was 60 µL. This is consistent with the concept of green chemistry, which recommends using as little solvents as possible. The US Environmental Protection Agency (EPA) has classified chlorobenzene as substance causing acute toxicity, histopathological changes and carcinogenicity [42]. Nevertheless, the use of a very small volume of chlorobenzene for the extraction minimizes the risk of exposure to this potentially hazardous solvent.

#### 3.2.2. Effect of Derivatization Reagent Volume

Bisphenols are compounds with high boiling points and low volatility, which limits their determination by the gas chromatography technique. Acetic anhydride, unlike silylation, which must be carried out as a separate step in the procedure, enables simultaneous derivatization and extraction in the sample matrix [23,43,44,45,46]. Carrying out derivatization simultaneously with emulsification is advantageous because of the simplicity of execution and the shortening of the sample preparation time. This allows for quick results and potential application in routine environmental testing. The results show that the optimal volume of the anhydride was 225 µL, so this volume was used for further experiments.

#### 3.2.3. The Influence of Salt

In general, the addition of salt in the extraction process increases the concentration of the analyte in the organic phase through the salting-out effect. Disodium hydrogen phosphate was used to evaluate the effect of salt on the separation efficiency of BPs. The higher the salt concentration used, the greater the surface area of the BPs obtained. In the absence of salt, the extraction efficiency of bisphenols is lower, whereas an addition of 4% disodium hydrogen phosphate results in the highest SN ratio and, in turn, extraction efficiency. 

### 3.3. Effect of Simultaneous Extraction and Derivatization Time

**Figure 3:**
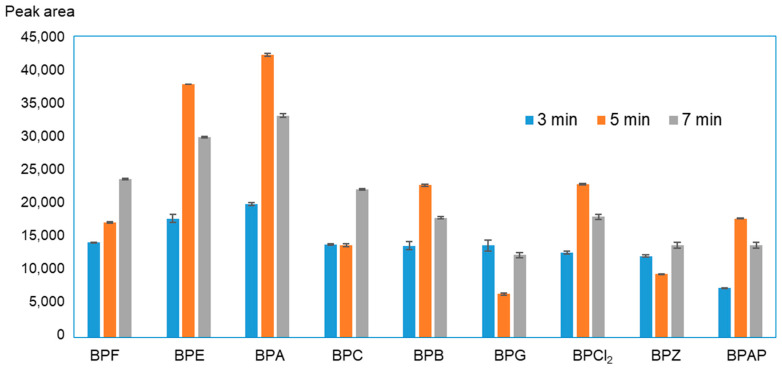
Influence of emulsification time on BP extraction efficiency.

The optimal extraction time was determined by carrying out the process under optimal conditions, changing the mixture of analytes with extractant and derivatizing reagent contact time with ultrasound. Results shown in Figure 3 indicate that the BP extraction process is most effective when the aqueous and organic phases are in contact for 5 min. Excessive ultrasound exposure reduces the efficiency of derivatization by hydrolysis of the resulting acylated derivatives. A literature review showed that in the case of other compounds isolated by the USAEME technique, a simultaneous extraction and derivatization time of 5 min provides the best results [47].

### 3.4. Method Validation Parameters

The analytical parameters of linearity, precision, LoD, LoQ and recovery were determined under optimal conditions. Before determination by GC-MS, the calibration curves were obtained by adding a mixture of standard solutions at concentration levels ranging from 0.005 to 500 μg/L to ultrapure water and then conducting extraction. Validation parameters of the USAEME-GC/MS procedure for BP determination are summarized in Table 5. The calibration curves were linear in the concentration ranges given for all compounds, and the coefficients of determination were R^2^ ≥ 0.997. The LoD was established as the concentration that resulted in a signal-to-noise ratio (S/N ratio) of 3. The concentrations corresponding to the lowest points of the calibration plots were established as the LoQ values. LoD values for the tested BPs ranged from 0.01 to 19.3 ng/L. The lowest LoDs were recorded for BPG, BPE and BPF. The precision of the method was evaluated using coefficient of variation (CV) values. The CV was calculated as the ratio of the method standard deviation to the mean value of the method. For individual compounds, mean CV values ranging from 2.16 to 8.59% were obtained. As expected, the measurements for higher concentrations are characterized by the highest precision. Recoveries for each compound were determined at two concentration levels: 1 and 10 μg/L. They were calculated by comparing nominal concentration with the value determined based on the calibration curve. The recovery values were between 92 and 122% for the lower concentration and between 88 and 113% for the higher value.

### 3.5. Matrix Effect

The developed method was also validated on two real matrices. The main pollution parameters characterized by the tested wastewater are summarized in Table 6. 

USAEME-GC/MS determinations were carried out for wastewater spiked with the determined compounds in various concentrations. From this, calibration curves were plotted, and validation parameters were determined. Because the signals corresponding to the tested compounds were detected in the selected matrices, the signals recorded for the same retention times during the preparation of the methodological blanks were subtracted from the signals recorded for spiked matrices. The validation parameters obtained for real matrices are presented in Table 7. For these two matrices, good linearity, expressed as an R^2^ value above 0.997, was obtained. The recovery values ranged from 85 to 119% for raw wastewater and from 87 to 122% for treated wastewater. This means they did not differ significantly from the values determined when ultrapure water was used as the sample matrix. CV values did not exceed 7.5% in either matrix. The mean sensitivity of BP determination expressed by the LoD value ranged from 4.16 to 63.82 ng/L when raw wastewater was used as a sample matrix and from 0.4 to 14.6 ng/L when treated wastewater was used. In the case of raw wastewater as a sample matrix, the deviation of validation parameters from those registered for water was greater than that of treated sewage. The mean CV value is about 0.2 times higher for raw wastewater (CV = 5.07%) than for water (CV = 4.22%) and slightly higher for treated wastewater (CV = 4.36%) than for water. Significant differences were observed when comparing the average LoD value for raw wastewater (LoD = 16.87 ng/L) with the average LoD value for water (LoD = 6.94 ng/L), as this value is almost 2.5 times higher for wastewater than for water. Measurements of pH, conductivity, BOD, COD, nitrogen and phosphorus concentrations showed the dependence between the deterioration of recovery and reduction in sensitivity and higher contamination of the matrix expressed. The obtained results indicate that the matrices have a slight but important influence on the validation parameters. The reason for this influence is predominately the change in the partition coefficient values of the tested compounds in the water/chlorobenzene system related to the presence of various organic and inorganic compounds in the wastewater. 

### 3.6. Comparison of the Developed USAEME-GC/MS Procedure with Other BP Assay Procedures

Table 8 compares the validation parameters of the developed USAEME-GC/MS method with methods of BP determination in different matrices described in the literature. The comparison with other methods based on the use of the GC/MS technique shows that depending on the matrix under consideration, the quality of the proposed method is similar or even higher [29,48,49,50]. When we consider the same matrices, i.e., wastewater, we can see that in terms of precision, accuracy and repeatability, the developed method does not differ from the methods described in the literature [46,48]. This is a satisfactory result, considering that the procedure using USAEME for isolation is not only much shorter and simpler but also less costly and more environmentally friendly. The comparison of the proposed USAEME-GC/MS method with the methods based on the use of LC-MS/MS also shows favorable results, taking into account the validation parameters. Slightly better sensitivity and determination accuracy are achieved by combining LC with a fluorescence detector (FLD). However, the cost of the assays and the availability of LC-MS/MS and LC-FLD instruments remain an issue.

### 3.7. Wastewater Analysis

**Figure 4:**
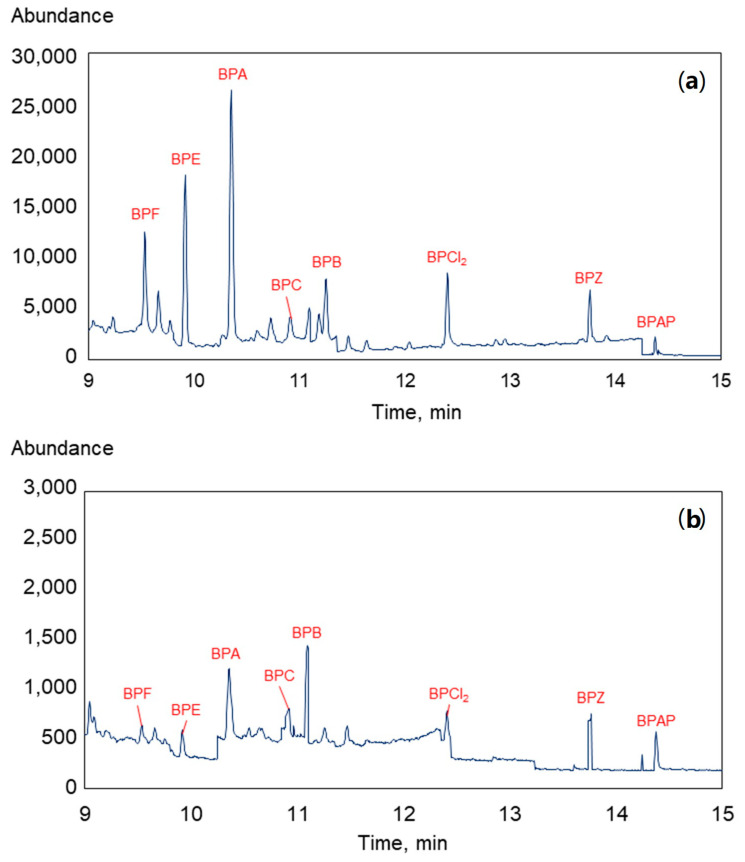
Chromatograms of raw (**a**) and treated (**b**) wastewater samples.

The developed USAEME-GC-MS method was used for the simultaneous determination of nine BPs in raw and treated wastewater samples. Figure 4 shows the chromatograms recorded for both types of wastewater. 

For BP determination in raw and treated wastewater, the calibration curve registered in these matrices were used. Table 9 summarizes the results of the conducted analysis, as well as the removal efficiency (RE) of individual compounds in the technological process in the wastewater treatment plant. As expected, the concentrations of bisphenols in raw wastewater are higher than those after the wastewater treatment process. The highest concentration (about 399 ng/L) was recorded for BPA, which is known to be the most common BP in industry and in the environment. After the purification process, its concentration decreases by about 75%. The concentration of BPA registered in other studies in influent wastewater varies widely, from a few ng/L to a dozen µg/L [22,34]. Usually, in areas with higher population densities and more developed industries, the BPA content is very high [32,33]. The second most abundant compound determined in the analyzed samples is BPZ (approximately 67 ng/L), with two-thirds of this compound removed from wastewater in the treatment process. BPF, which is one of the most commonly used BPA substitutes, is removed in over 88%. Its concentration decreases from almost 40 ng/L to a concentration below the LoD. Concentrations of BPC and BPG in raw wastewater are below the LoD (15.33 ng/L and 9.12 ng/L for BPC and BPG, respectively). However, the effluent concentration of BPC is 7.57 ng/L, and that of BPG is 33.08 ng/L. Despite the lack of a determined concentration in the influent, it can be concluded that these two BPs can undergo sorption processes on particles suspended in the wastewater matrix and desorb during treatment, which may contribute to changes in concentrations. The concentration of BPE and BPZ is 58.71 and 66.63 ng/L, respectively. Česen et al. reported that these BPs are dominant compounds in the influent from a Slovenian WWTP (concentration of BPE and BPZ: 238 ng/L and 403 ng/L, respectively) [48]. The BPZ removal efficiency is 57%, which is close to values in the literature data. Only about 7% of BPCl_2_ is removed in the technological process of wastewater treatment. The lack of literature data on BPCl_2_ concentrations in water matrices does not allow for precise determination of the removal mechanism of this compound. 

## 4. Conclusions

A new procedure for simultaneous extraction using the USAEME technique and for determination by GC-MS of nine analogs of bisphenols was proposed. The extraction procedure was carried out simultaneously with the derivatization step. We found that the extraction efficiency was influenced by the type and volume of solvent used, the amount of derivatizing reagent, the salt concentration and the contact time of the extractant with the sample. Optimal parameters were selected using the Taguchi experimental method. The proposed model explained as much as 80% of the variability, which proves that it is a good fit. Based on the optimization step, 60 µL of chlorobenzene, 225 µL of acetic anhydride and 4% salt with an emulsification time of 5 min were selected as optimal. The developed procedure for the extraction and determination of bisphenols was fully validated, allowing for determination of the examined bisphenols in the range of 0.005–500 µg/L, with recovery ranging from 92 to 122% for a concentration of 1 µg/L and 88–113% for a concentration of 10 ug/L. The obtained values of LoD lead to the conclusion that the elaborated procedure is suitable for testing the content of BPs in environmental samples.

## Figures and Tables

**Table 1:** Basic information about determined bisphenols.

Compound	Abbreviation/CAS	Structure	MM (g/mol)	logK_ow_	pK_a_	t_R_ (min)	Characteristic Masses
Bisphenol F (4,4′-Methylenediphenol)	BPF 1333-16-0	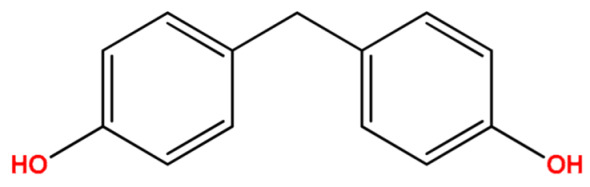	200.23	2.76	9.91	9.56	107, 183, **200**
Bisphenol E (4,4′-Ethylidenebisphenol)	BPE 2081-08-5	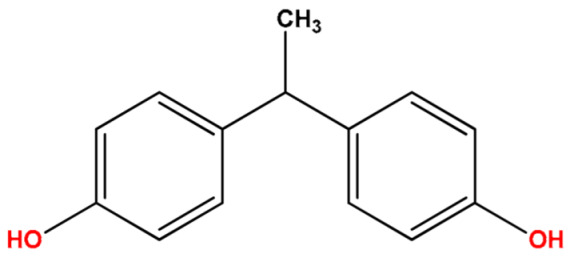	214.26	3.23	10.10	9.94	181, **199**, 214
Bisphenol A (4,4′-Isopropylidenediphenol)	BPA 80-05-7	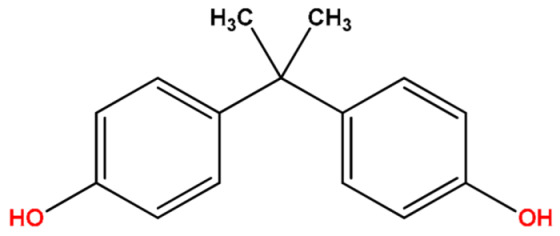	228.11	3.64	10.29	10.38	119, **213**, 228
Bisphenol C (4,4′-Isopropylidenedi-*o*-cresol)	BPC 79-97-0	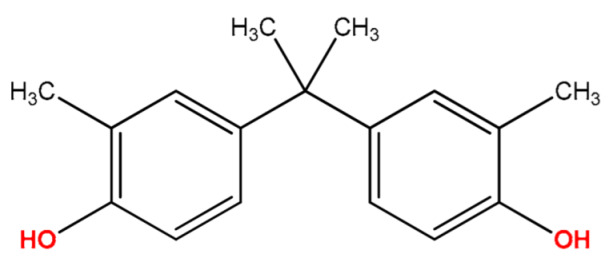	256.34	4.74	9.90	10.94	133, **241**, 256
Bisphenol B (4,4′-*sec*-Butylidenediphenol)	BPB 77-40-7	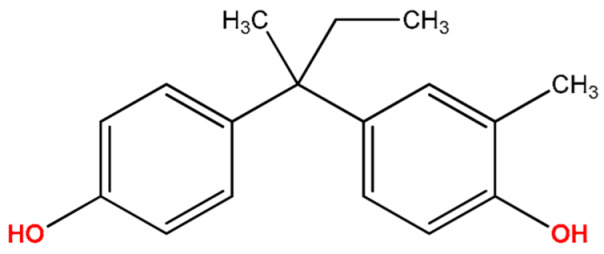	242.32	4.15	10.27	11.29	119, **213**, 242
Bisphenol G (4,4′-Isopropylidenebis(2-isopropylphenol))	BPG 125-54-8	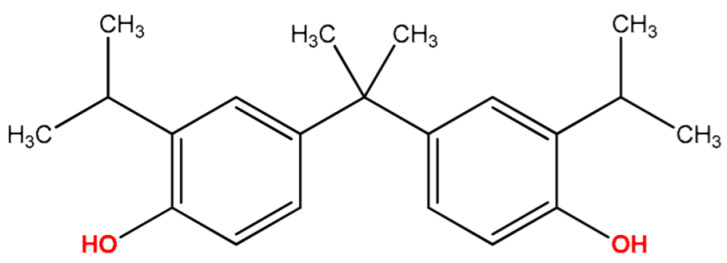	312.45	6.55	–	11.49	177, **297**, 312
Bisphenol Cl_2_ (4,4′-(2,2-dichloroethene-1,1-diyl)diphenol)	BPCl_2_ 14868-03-2	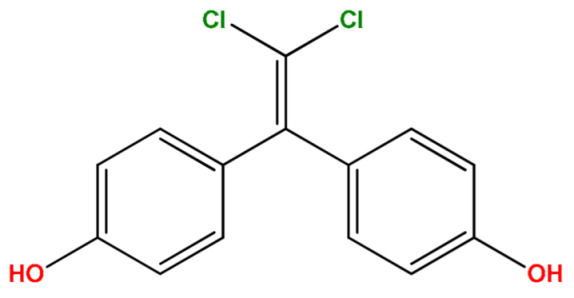	281.13	–	–	12.42	152, 210, **280**
Bisphenol Z (4,4′-Cyclohexylidenebisphenol)	BPZ 843-55-0	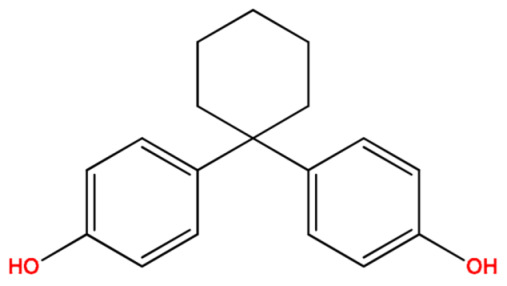	268.36	4.87	9.91	13.77	199, 225, **268**
Bisphenol AP (4,4′-(1-Phenylethylidene)bisphenol)	BPAP 1571-75-1	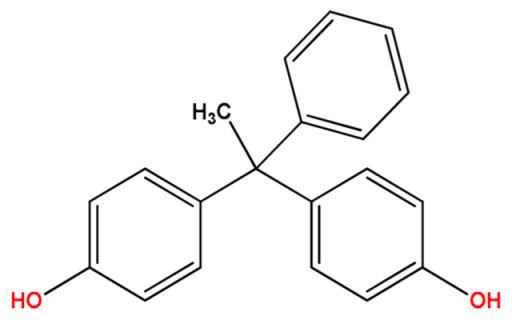	290.36	4.33	10.22	14.39	**275**, 276, 290

MM—molar mass, logK_ow_—octanol/water partition coefficient, pK_a_—acid dissociation constant, t_R_—retention time.

**Table 2:** Taguchi orthogonal array for BPA.

Run	Chlorobenzene Volume (μL)	Acetic Anhydride Volume (μL)	Salt Concentration (%)	Mean Peak Area	SN Ratio
1	60	125	0	12,155,020	141.7
2	60	175	2	36,898,612	151.3
3	60	225	4	42,227,803	152.5
4	80	125	2	26,066,185	148.3
5	80	175	4	28,628,198	149.1
6	80	225	0	11,275,386	141.0
7	100	125	4	24,357,492	147.7
8	100	175	0	7,285,741	137.2
9	100	225	2	19,889,630	146.0

**Table 3:** Analysis of variance for SN ratios.

Source	DF	Seq SS	Adj SS	Adj MS	F	P	Percentage Contribution
PhCl	2	35.505	35.505	17.7523	19.09	0.050	17.01
Ac_2_O	2	0.712	0.712	0.3559	0.38	0.723	0.34
Salt	2	170.641	170.641	85.3206	91.76	0.011	81.75
Residual Error	2	1.860	1.860	0.9298			0.9
Total	8	208.717					100
Model summary	S = 0.9643						
	R^2^ = 96.30%						
	Adjusted R^2^ = 85.19%						

**Table 4:** Comparison of predicted and experimental SN ratios.

Compound	Predicted SN Ratio	Experimental SN Ratio
BPF	149.8	147.5
BPE	151.8	149.5
BPA	152.6	150.4
BPC	144.8	146.9
BPB	147.1	145.0
BPG	141.3	141.7
BPCl_2_	147.1	145.2
BPZ	144.8	142.8
BPAP	144.8	142.8

**Table 5:** USAEME–GC–MS method validation parameters determined with water as a sample matrix.

Compound	Linearity			R^2^	Recovery (%)		CV (%)	LoD (ng/L)
	Range (μg/L)	Slope	Intercept		1 μg/L	10 μg/L		
BPF	0.005–500	308,626	1,149,749	0.998	92 ± 5	97 ± 3	2.91	2.23
BPE	0.005–500	371,209	1,385,372	0.997	95 ± 2	103 ± 1	3.02	1.48
BPA	0.05–500	400,389	1,355,027	0.998	95 ± 4	99 ± 2	3.01	5.85
BPC	0.05–500	265,935	753,368	0.998	107 ± 2	105 ± 3	4.49	5.08
BPB	0.005–500	211,780	723,170	0.998	120 ± 4	112 ± 5	2.16	2.45
BPG	0.005–500	145,140	243,246	0.9993	108 ± 2	113 ± 2	8.59	0.06
BPCl_2_	0.05–500	223,987	177,362	0.9995	94 ± 4	90 ± 2	4.84	17.25
BPZ	0.05–500	158,799	75,071	0.9996	122 ± 9	89 ± 6	4.51	19.26
BPAP	0.05–500	153,658	286,635	0.9990	116 ± 3	88 ± 6	4.46	8.79

R^2^—coefficient of determination, CV—coefficient of variation, LoD—limit of detection.

**Table 6:** Physicochemical indicators of the tested wastewater.

Parameter	pH	σ (µS/cm)	COD (mg/L)	BOD (mg/L)	TN (mg/L)	TP (mg/L)
raw wastewater	8.23	1286.9	214	10	43.4	2.23
treated wastewater	7.82	961.4	31.3	4	10.8	0.143

σ—conductivity, COD—chemical oxygen demand, BOD—biochemical oxygen demand, TN—total nitrogen concentration, TP—total phosphorus concentration.

**Table 7:** USAEME–GC–MS method validation parameters determined with raw and treated wastewater as sample matrix.

Compound	Linearity			R^2^	Recovery (%)		CV (%)	LoD (ng/L)
	Range (μg/L)	Slope	Intercept		1 μg/L	10 μg/L		
**Raw wastewater**								
BP F	0.01–500	282,040	−1,338,731	0.9991	119 ± 5	112 ± 5	4.08	4.65
BP E	0.1–500	369,210	−680,445	0.9990	100 ± 8	98 ± 4	3.43	11.73
BP A	0.1–500	342,430	−302,379	0.9991	85 ± 2	101 ± 9	3.08	63.82
BP C	0.05–500	243,972	−1,054,776	0.998	86 ± 1	102 ± 6	6.50	15.33
BP B	0.01–500	230,244	−503,381	0.9994	118 ± 2	101 ± 3	5.87	4.16
BP G	0.05–500	142,492	−594,097	0.997	95 ± 7	102 ± 5	7.50	9.12
BP Cl_2_	0.05–500	207,697	−1,331,025	0.997	91 ± 3	115 ± 4	3.88	18.51
BP Z	0.05–500	197,901	−632,302	0.9993	114 ± 1	105 ± 6	5.81	8.16
BP AP	0.1–500	213,678	−462,226	0.9991	119 ± 3	103 ± 5	5.48	16.45
**Treated wastewater**								
BP F	0.005–500	240,799	−460,455	0.9994	116 ± 5	112 ± 3	2.93	4.57
BP E	0.05–500	357,110	−1,176,139	0.9997	106 ± 5	122 ± 3	2.73	4.69
BP A	0.05–500	339,543	−1,444,976	0.999	113 ± 4	113 ± 4	3.02	3.88
BP C	0.005–500	278,657	−877,267	0.999	108 ± 3	113 ± 1	4.28	0.41
BP B	0.05–500	231,149	−451,909	0.997	87 ± 7	115 ± 4	4.08	14.64
BP G	0.01–500	175,634	284,113	0.998	118 ± 2	94 ± 2	7.33	4.76
BP Cl_2_	0.05–500	178,346	−437,280	0.9995	99 ± 9	111 ± 2	4.60	5.18
BP Z	0.1–500	184,781	−937,391	0.998	106 ± 9	108 ± 5	4.91	4.63
BP AP	0.05–500	203,648	−532,571	0.998	118 ± 5	105 ± 4	5.40	11.72

R^2^—coefficient of determination, CV—coefficient of variation, LoD—limit of detection.

**Table 8:** Comparison of the validation parameters of the proposed method with literature data.

Compound	Method	Sample	Linear Range (μg/L)	LOD (ng/L)	Recovery (%)	Ref.
F, E, A, C, B, G, Cl_2_, Z, AP	USAEME-GC-MS	Raw wastewater	0.01–500	4.2–63,8	85–119	This study
		Treated wastewater	0.005–500	0.4–14.6	87–115	
F, A, Z, S	USAEME-GC-MS	Thermal paper, toys and baby utensils	0.1–3	10–30	-	[29]
F, E, A, C, Z, BP, S, FL, AF	SPE-GC-MS	Wastewater, surface water	0.0001–1	0.3–17	78–133	[49]
F, E, A, C, B, G, Cl_2_, Z, AP, S, M, BP, PH, TMC	SPE-GC-MS/MS	House dust	0.002–2.5 ^a^	1–17 ^a^	65–111	[50]
F, E, C, B, Z, AP, S, AF	SPE-GC-MS	Wastewater	0.004–1	0.207–1.20	79–100	[48]
F, E, A, S, F	SPE-LC-MS/MS	Wastewater	0.5–500	0.043–2.43	43–90	[22]
F, E, A, B, Z, AP, S, AF, TBBPA	SPE-HPLC-MS/MS	Wastewater	0.005–100	0.0007–16.3 ^b^	82–101	[21]
		Sludge		0.0004–8.28 ^a,b^	43–97	
F, A, BFDGE, BADGE	CPE-LC-DAD, FLD	Wastewater, river water	0.0001–0.05	9–10	95–102	[51]
F, A, C, B, AP, S, AF, TDP TBBPA, TCBPA, TMBPA,	UPLC-MS/MS	Bottled drinking water	0.01–200	0.01–100	75–102	[52]
F, A, S, AF, benzophenone	DLLME-UPLC-MS	Complex water matrices	0.50–200	0.05–0.1	60–120	[53]
F, A, S, AF, parabens	QuEChERS-LC-MS/MS	Breast milk	0.5–2000	10–200	77–98	[54]
A, C, B, Z, P, AP, AF, FL, TMBPA	HPLC-FLD	Children’s water bottles	0.0004–80	0.13–66.7	90–112	[55]

^a^—the value is given in ng/g; ^b^—LOQ.

**Table 9:** Bisphenol concentration (ng/L) in wastewater samples. SD calculated for three repetitions.

Compound	Raw Wastewater	Treated Wastewater	Removal Efficiency (%)
BPF	38.89 ± 0.51	<LoD	88.17
BPE	58.71 ± 3.82	25.16 ± 0.10	57.15
BPA	398.97 ± 9.24	101.84 ± 1.79	74.47
BPC	<LoD	7.57 ± 0.10	50.52
BPB	62.49 ± 4.27	29.29 ± 0.38	53.13
BPG	<LoD	33.08 ± 1.16	47.25
BPCl_2_	53.12 ± 1.63	49.54 ± 0.27	6.75
BPZ	66.62 ± 2.70	24.64 ± 0.26	63.01
BPAP	<LoD	<LoD	-

## Data Availability

Not applicable.

## Supplementary Materials

The following supporting information can be downloaded at: https://www.mdpi.com/article/10.3390/molecules27154977/s1, Figure S1: Chromatogram registered during GC-MS analysis of 500 μg/L bisphenol mixture in Milli-Q water; Figure S2: Mass spectra of acetylated bisphenol products registered in selected ion monitoring (SIM) mode; Tables S1–S9: Response tables for SNR of each controllable variable in the BP extraction process; Tables S10–S17: ANOVA for BP extraction optimization process.

## Sample Availability

Samples of the compounds used in experiments are available from the authors.
