# Supplementary material for: USAEME-GC/MS Method for Easy and Sensitive Determination of Nine Bisphenol Analogues in Water and Wastewater
Source: Molecules. 2022 Aug 5;27(15):4977. doi: 10.3390/molecules27154977 (PMC9370219; doi:10.3390/molecules27154977)
Supplement: Supplementary file 1 [file molecules-27-04977-s001.zip › molecules-1808998-supplementary.pdf]

molecules

MDPI

Supplementary Materials

USAEME-GC/MS method for easy and sensitive determination of nine bisphenol analogues in water and wastewater

Dariusz Kiejza[1], Weronika Polińska[1], Joanna Karpińska[2] and Urszula Kotowska[2,*]

1 Doctoral School of Exact and Natural Sciences, University of Bialystok, Ciołkowskiego 1K St., 15-245 Białystok, Poland; d.kiejza@uwb.edu.pl, w.polinska@uwb.edu.pl
2 Department of Analytical and Inorganic Chemistry, Faculty of Chemistry, University of Bialystok, Ciołkowskiego 1K St., 15-245 Białystok, Poland; joasia@uwb.edu.pl
*Correspondence: ukrajew@uwb.edu.pl; Tel.: +48-85-738-8111

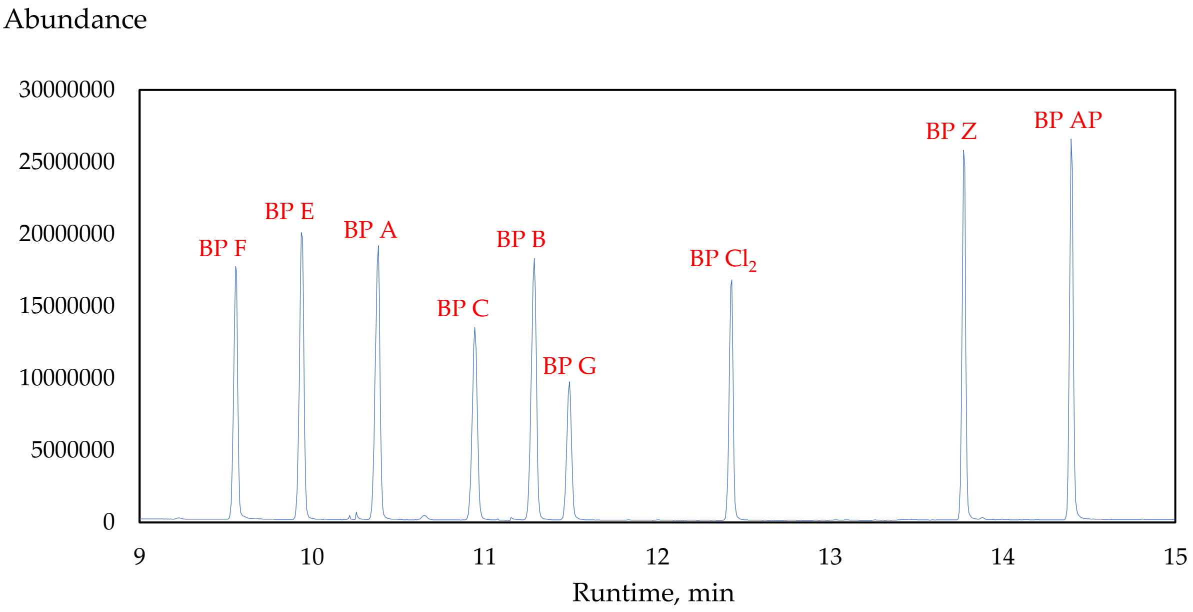

Figure S1. Chromatogram registered during GC-MS analysis of 500ug/L bisphenols mixture in Milli-Q water.

Bisphenol F

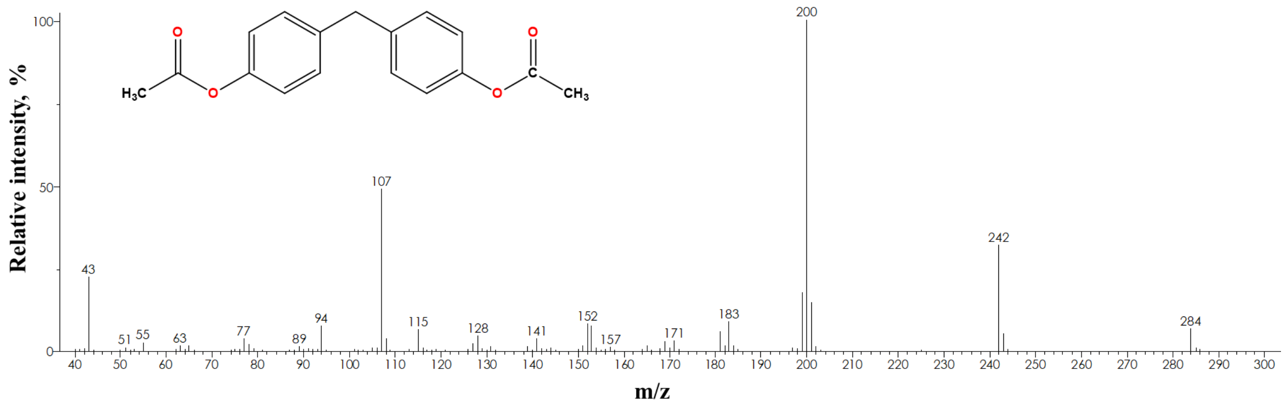

Bisphenol E

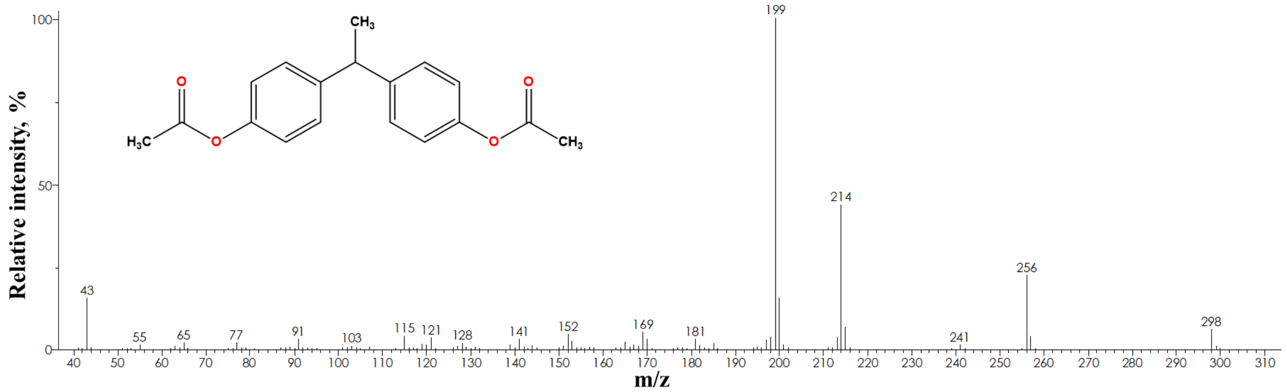

Bisphenol A

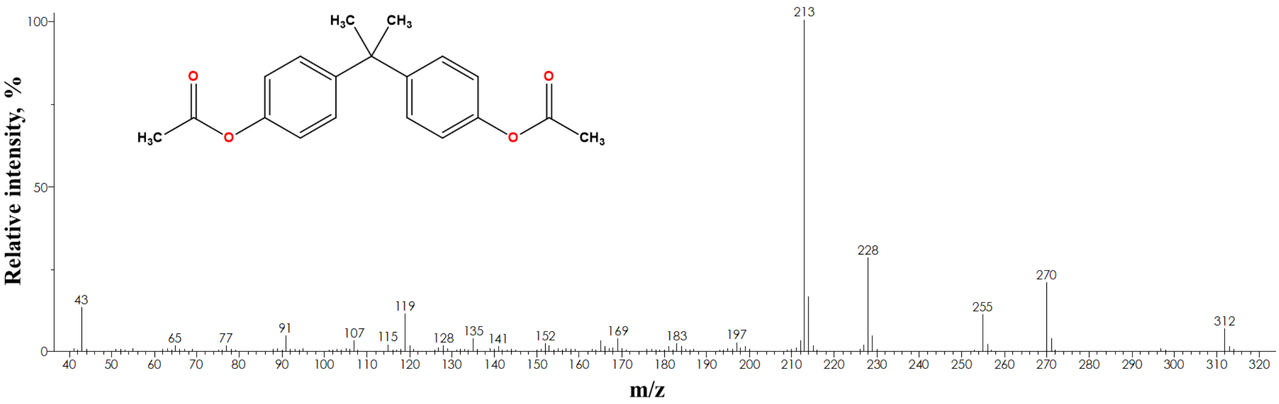

Bisphenol C

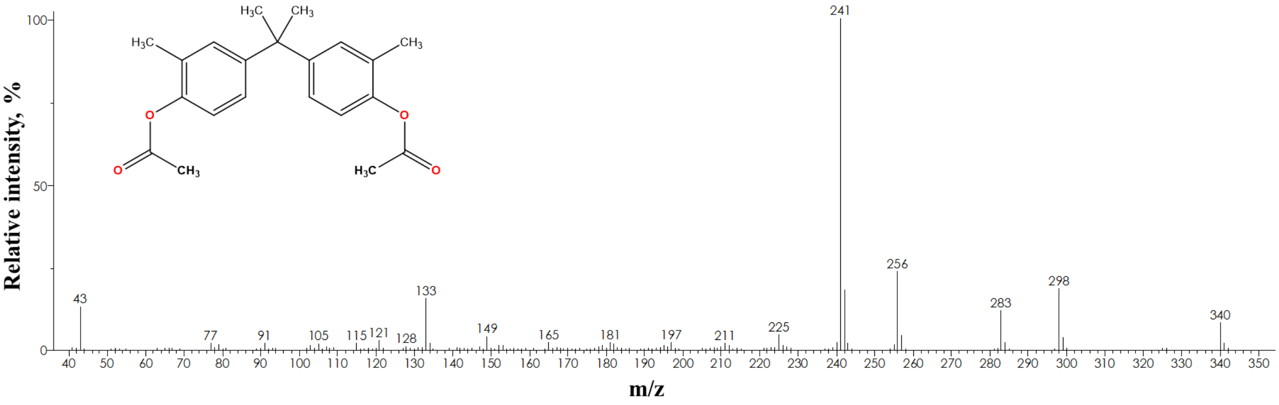

Bisphenol B

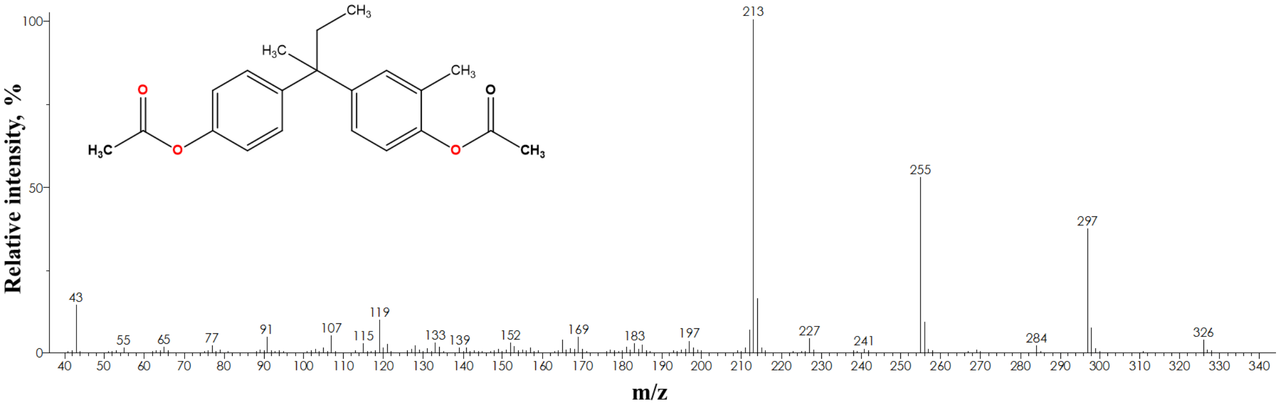

Bisphenol G

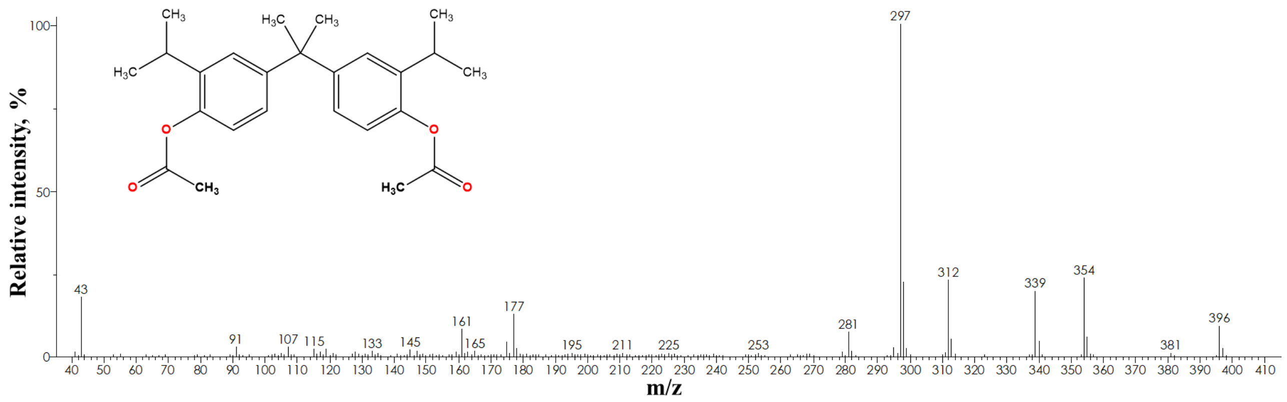

Bisphenol Cl2

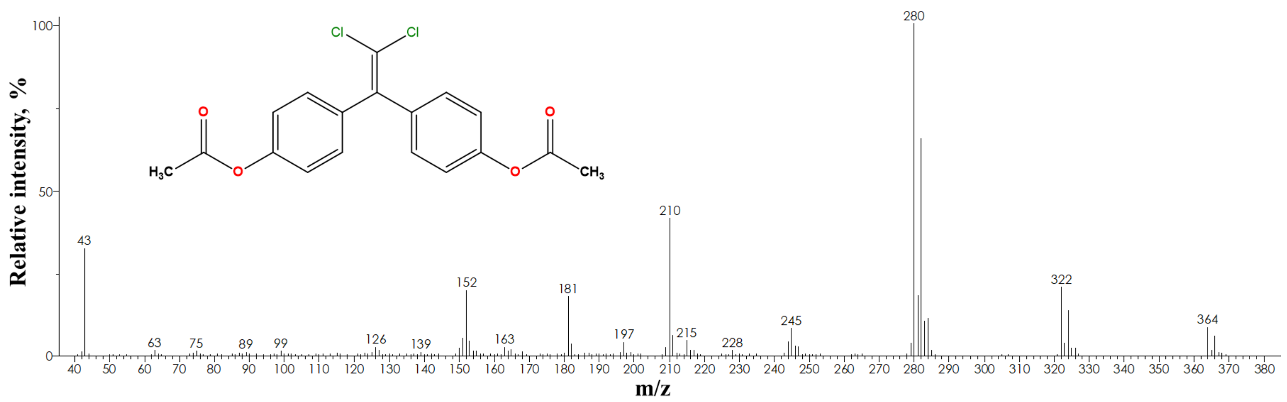

Bisphenol Z

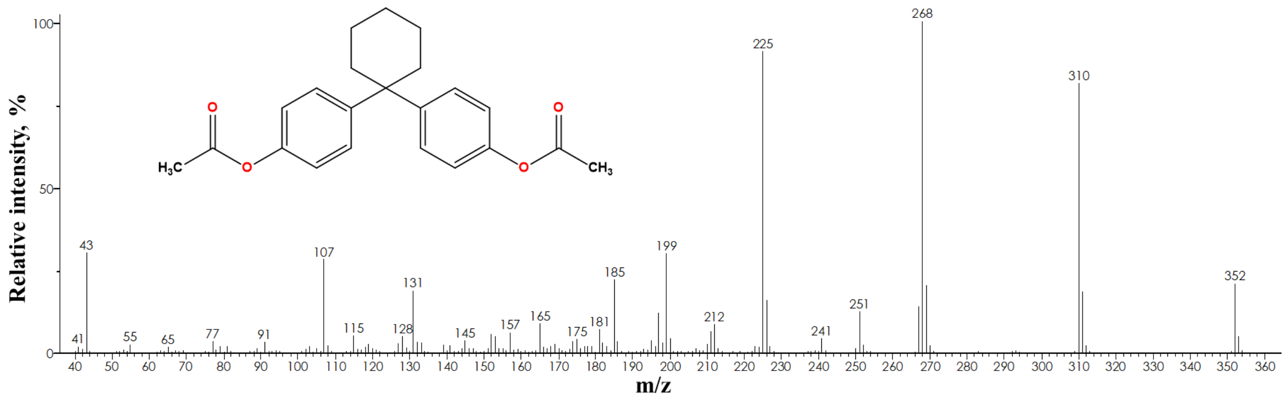

Bisphenol AP

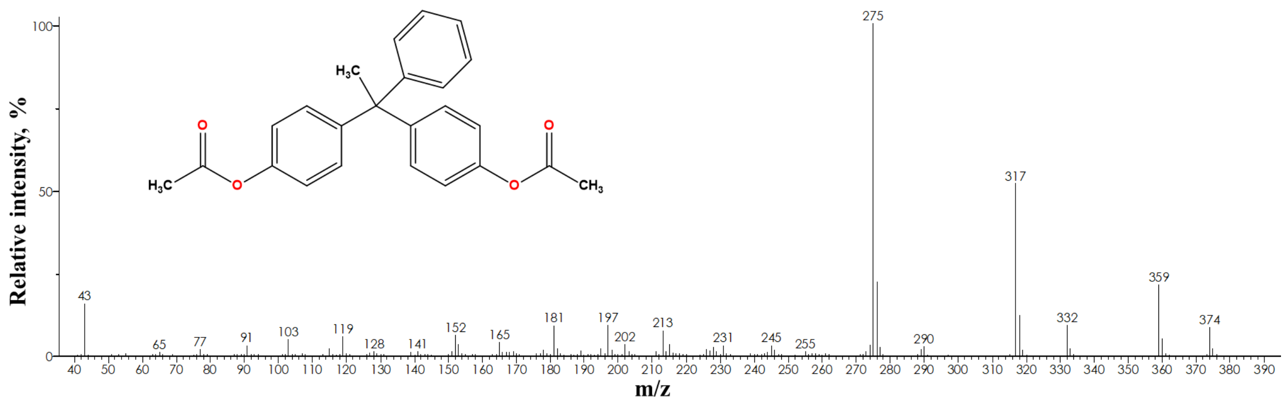

Figure S2. Mass spectra of acetylated bisphenols products registered in selected ions monitoring (SIM) mode.

Table S1. Response table for SNR of each controllable variables in BPF extraction process.

Level	Chlorobenzene	Acetic anhydride	Salt
1	141.0	138.4	124.1
2	139.4	138.4	145.5
3	136.1	139.6	146.8
Delta	4.9	1.3	22.7
Rank	2	3	1

Table S2. Response table for SNR of each controllable variables in BPE extraction process.

Level	Chlorobenzene	Acetic anhydride	Salt
1	145.7	143.1	133.6
2	143.6	143.0	147.5
3	140.7	143.9	148.9
Delta	5.0	0.9	15.3
Rank	2	3	1

Table S3. Response table for SNR of each controllable variables in BPA extraction process.

Level	Chlorobenzene	Acetic anhydride	Salt
1	148.5	145.9	140.0
2	146.2	145.9	148.5
3	143.7	146.5	149.8
Delta	4.9	0.6	9.8
Rank	2	3	1

Table S4. Response table for SNR of each controllable variables in BPC extraction process.

Level	Chlorobenzene	Acetic anhydride	Salt
1	147.6	144.4	145.3
2	145.1	145.7	144.7
3	143.0	145.6	145.7
Delta	4.6	1.4	1.0
Rank	1	2	3

Table S5. Response table for SNR of each controllable variables in BPB extraction process.

Level	Chlorobenzene	Acetic anhydride	Salt
1	144.8	142.2	139.9
2	142.4	142.5	143.0
3	140.2	142.7	144.5
Delta	4.7	0.5	4.6
Rank	1	3	2

Table S6. Response table for SNR of each controllable variables in BPG extraction process.

Level	Chlorobenzene	Acetic anhydride	Salt
1	142.6	139.9	142.5
2	140.7	141.1	140.0
3	138.3	140.6	139.1
Delta	4.4	1.2	3.4
Rank	1	3	2

Table S7. Response table for SNR of each controllable variables in $BPCl_2$ extraction process.

Level	Chlorobenzene	Acetic anhydride	Salt
1	145.5	142.8	141.6
2	142.8	142.9	142.8
3	140.6	143.2	144.4
Delta	4.9	0.4	2.8
Rank	1	3	2

Table S8. Response table for SNR of each controllable variables in BPZ extraction process.

Level	Chlorobenzene	Acetic anhydride	Salt
1	143.0	140.3	139.0
2	140.5	140.6	140.4
3	138.2	140.7	142.2
Delta	4.7	0.4	3.2
Rank	1	3	2

Table S9. Response table for SNR of each controllable variables in BPAP extraction process.

Level	Chlorobenzene	Acetic anhydride	Salt
1	142.9	140.6	139.6
2	140.7	140.4	140.1
3	138.4	141.0	142.3
Delta	4.5	0.5	2.6
Rank	1	3	2

Table S10. ANOVA for BPF extraction optimization process.

Source	DF	Seq SS	Adj SS	Adj MS	F	P	Percentage contribution
PhCl	2	37,02	37,017	18,508	8,93	0,101	3,64
Ac_2O	2	3,05	3,049	1,524	0,74	0,576	0,30
Salt	2	972,17	972,172	486,086	234,63	0,004	95,65
Residual Error	2	4.14	4.143	2.072			0,41
Total	8	1016.38					100
		**S= 1.4394**					
Model summary		**R^2= 99,59 %**					
		**Adjusted R^2= 98.37 %**					

Table S11. ANOVA for BPE extraction optimization process.

Source	DF	Seq SS	Adj SS	Adj MS	F	P	Percentage contribution
PhCl	2	37,693	37,693	18,847	14,05	0,066	8,01
Ac_2O	2	1,479	1,479	0,740	0,55	0,645	0,31
Salt	2	428,662	428,662	214,331	159,79	0,006	91,10
Residual Error	2	2,683	2,683	1,341			0,58
Total	8	470,518					100
		**S= 1.1582**					
Model summary		**R^2= 99,43 %**					
		**Adjusted R^2= 97.72 %**					

Table S12. ANOVA for BPC extraction optimization process.

Source	DF	Seq SS	Adj SS	Adj MS	F	P	Percentage contribution
PhCl	2	31,6884	31,6884	15,8442	88,11	0,011	85,69
Ac_2O	2	3,4564	3,4564	1,7282	9,61	0,094	9,35
Salt	2	1,4756	1,4756	0,7378	4,10	0,196	3,99
Residual Error	2	0,3596	0,3596	0,1798			0,97
Total	8	36,9800					100
		**S= 0.4240**					
Model summary		**R^2= 99,03 %**					
		**Adjusted R^2= 96.11 %**					

Table S13. ANOVA for BPB extraction optimization process.

Source	DF	Seq SS	Adj SS	Adj MS	F	P	Percentage contribution
PhCl	2	32,6099	32,6099	16,3050	26,89	0,036	48,25
Ac_2O	2	0,4205	0,4205	0,2102	0,35	0,743	0,62
Salt	2	33,3456	33,3456	16,6728	27,49	0,035	49,33
Residual Error	2	1,2129	1,2129	0,6064			1,8
Total	8	67,5889					100
		**S= 0.7787**					
Model summary		**R^2= 98.21 %**					
		**Adjusted R^2= 92.82 %**					

Table S14. ANOVA for BPG extraction optimization process.

Source	DF	Seq SS	Adj SS	Adj MS	F	P	Percentage contribution
PhCl	2	29,150	29,150	14,575	14,22	0,066	56,37
Ac_2O	2	2,017	2,017	1,009	0,98	0,504	3,90
Salt	2	18,491	18,491	9,245	9,02	0,100	35,76
Residual Error	2	2,050	2,050	1,025			3,97
Total	8	51,708					100
Model summary		**S= 1.0124**<br>**R^2= 96.04 %**<br>**Adjusted R^2= 84.14 %**					

Table S15. ANOVA for $BPCl_2$ extraction optimization process.

Source	DF	Seq SS	Adj SS	Adj MS	F	P	Percentage contribution
PhCl	2	35,9486	35,9486	17,9743	42,86	0,023	74,01
Ac_2O	2	0,2181	0,2181	0,1091	0,26	0,794	0,45
Salt	2	11,5652	11,5652	5,7826	13,79	0,068	23,81
Residual Error	2	0,8388	0,8388	0,4194			1,73
Total	8	48,5707					100
Model summary		**S= 0.6476**<br>**R^2= 98.27 %**<br>**Adjusted R^2= 93.09 %**					

Table S16. ANOVA for BPZ extraction optimization process.

Source	DF	Seq SS	Adj SS	Adj MS	F	P	Percentage contribution
PhCl	2	33,8580	33,8580	16,9290	28,26	0,034	66,95
Ac_2O	2	0,2544	0,2544	0,1272	0,21	0,825	0,50
Salt	2	15,2605	15,2605	7,6303	12,74	0,073	30,18
Residual Error	2	1,1983	1,1983	0,5992			2,37
Total	8	50,5713					100
Model summary		**S= 0.7740**<br>**R^2= 97.63 %**<br>**Adjusted R^2= 90.52 %**					

Table S17. ANOVA for BPAP extraction optimization process.

Source	DF	Seq SS	Adj SS	Adj MS	F	P	Percentage contribution
PhCl	2	30,1033	30,1033	15,0516	46,96	0,021	69,74
Ac_2O	2	0,4197	0,4197	0,2099	0,65	0,604	0,97
Salt	2	12,0030	12,0030	6,0015	18,73	0,051	27,80
Residual Error	2	0,6410	0,6410	0,3205			1,49
Total	8	43,1670					100
Model summary		**S= 0.5661**<br>**R^2= 98.52 %**<br>**Adjusted R^2= 94.06 %**					
